# A systematic critical review on MRI in spondyloarthritis

**DOI:** 10.1186/ar3768

**Published:** 2012-03-09

**Authors:** Bodil Arnbak, Charlotte Leboeuf-Yde, Tue Secher Jensen

**Affiliations:** 1 Research Department, Spine Centre of Southern Denmark, Hospital Lillebaelt, Clinical Locomotion Network, Oestre Hougvej 55, Middelfart 5500, Denmark; 2 Institute of Regional Health Services Research, University of Southern Denmark,Winsloewparken 19, Odense C 5000, Denmark

## Abstract

**Introduction:**

Magnetic resonance imaging (MRI) has been proven capable of showing inflammatory and structural changes in patients with spondyloarthritis (SpA) and has become widely used in the diagnosis of SpA. Despite this, no systematic reviews evaluate the diagnostic utility of MRI for SpA. Therefore, the objective of this systematic review was to determine the evidence for the utility of MRI in the clinical diagnosis of SpA. The aims were to identify which MRI findings are associated with the diagnosis of SpA and to quantify this association.

**Methods:**

MEDLINE and EMBASE were electronically searched. Inclusion criteria were cross-sectional or longitudinal case-control or cohort MRI studies. The studies required a group with either SpA or inflammatory back pain (IBP) and a non-case group without SpA or IBP. Each group required a minimum of 20 participants. The included articles had to report results containing raw numbers suitable for the construction of two-by-two tables or report results by sensitivity and specificity for cross-sectional studies or odds ratios, relative risk ratios, or likelihood ratios for longitudinal studies. Method quality was assessed by using criteria based on the QUADAS tool.

**Results:**

In total, 2,395 articles were identified in MEDLINE and EMBASE before November 2011. All articles were reviewed by title and abstract. Seventy-seven articles were reviewed by full text, and 10 met the inclusion criteria. Two were considered of high quality: one evaluated the sacroiliac joints, and the other, the spine. Because of the small number of high-quality studies, a meta-analysis was not performed. The two high-quality studies found a positive association between MRI findings (bone marrow edema, erosions, fat infiltrations, global assessment of sacroiliitis, and ankylosis) and the diagnosis of IBP and SpA.

**Conclusion:**

In this review, several MRI findings were found to be associated with SpA. However, because of the small number of high-quality studies, the evidence for the utility of MRI in the diagnosis of SpA must be considered limited. Therefore, caution should be taken to ensure that inflammatory and structural MRI findings are not interpreted as being more specific for SpA than is supported by research.

## Introduction

Spondyloarthritis (SpA) is a rheumatologic disease that comprises the different disease entities of ankylosing spondylitis (AS), reactive arthritis, psoriasis arthritis, spondyloarthritis associated with inflammatory bowel disease, and undifferentiated spondyloarthritis. The main symptoms are chronic back pain, associated with extraspinal manifestations and particular laboratory findings. It usually emerges in the second or third decade of life and, although it is relatively rare, the prevalence being about 0.25% to 1% in the European population [1], it is a health condition worthy of attention because it can be debilitating for those affected, especially if undiagnosed.

With the introduction of tumor necrosis factor-α inhibitors in the treatment of preradiographic SpA [2,3], the interest in early and precise diagnosis has increased. However, the early diagnosis of SpA is often difficult. In chronic back pain patients, the most common diagnosis is nonspecific low-back pain (NSLBP), and SpA is estimated to contribute only 5% of the causes of back pain in primary care patients [4]. Even though plain radiography can detect structural changes of the spine and sacroiliac joints (SIJs), patients often have symptoms for several years before these changes become evident [5].

Over the past three decades, magnetic resonance imaging (MRI) has proven capable of detecting preradiographic inflammatory lesions seen in SpA patients [6,7], and optimism exists regarding the opportunities MRI can offer for early diagnosis of SpA. This is indicated by the inclusion of MRI of the SIJ in the criteria for axial SpA developed by the Assessment of SpondyloArthritis international Society (ASAS) in 2009 [8]. However, to our knowledge, no systematic critical literature reviews have addressed the utility of MRI in the diagnosis of SpA.

The objective of this systematic critical review was therefore to determine the level of evidence for the utility of MRI in relation to the clinical diagnosis of SpA. The specific aims of this review were to identify which MRI findings are associated with the diagnosis of SpA and to quantify this association.

## Methods

### Search method for identification of studies

Searches were made in the MEDLINE and EMBASE databases for articles published before November 2011. The following search terms were used as free text and MeSH terms: "magnetic resonance imaging," "spondyloarthritis," "ankylosing spondylitis," "sacroiliitis," "psoriasis arthritis," "reactive arthritis," "arthritis and inflammatory bowel disease," and "inflammatory back pain." Different forms of spelling and synonyms for each term were also used.

### Inclusion criteria for considering studies for this review

#### Types of studies

Prospective and retrospective case-control or cohort studies were accepted. Both cross-sectional and longitudinal studies were accepted. Articles in English, German, French, Norwegian, Swedish, and Danish were included, as these were the linguistic capabilities of the author team.

#### Study subjects

Studies were required to include a group of cases with the clinical diagnosis of SpA or inflammatory back pain (IBP) and a group of noncases without SpA or IBP, respectively. Each case and noncase group had to contain more than 20 participants. The criteria for sample size were set arbitrarily to reduce the risk of imprecise estimates with very wide confidence intervals (CIs). The mean age of the study sample had to be older than 18 and younger than 65 years.

#### Index test

The index test under evaluation was MRI of the axial skeleton. The field strength had to be a minimum of 1.5 Tesla to secure a minimal standard of the imaging. No other limitations were set for the technical equipment used, and all types of MRI sequences were accepted.

#### Target condition

The target condition was either SpA, one of the disease subgroups of SpA (AS, psoriasis arthritis, reactive arthritis, arthritis associated with IBP, or undifferentiated SpA) or IBP.

#### Reference standard

The reference standard was the clinical diagnosis of one of the target conditions defined by a diagnostic criterion or by expert opinion. The diagnosis had to have been made from clinical information. Information from plain radiography and serologic testing was accepted as part of the diagnosis.

#### Results presentation

Articles were included only if they reported one of the following: Results containing raw numbers suitable for the construction of two-by-two tables, for cross-sectional studies if they reported results by sensitivity and specificity, and for longitudinal studies if they reported odds ratios, relative risk ratios, or likelihood ratios.

### Data collection and analysis

#### Selection of studies

Initially, the first author reviewed the titles and abstracts of the search results for identification of relevant articles to be retrieved in full text. The full texts were screened for relevance, and double publications were excluded at this stage. Subsequently, the identified articles were reviewed in full text for data extraction by the first and third authors.

#### Data extraction and management

The first and third authors independently extracted the data from the relevant articles according to a check-list specifying the information needed regarding the following factors: (a) study sample(s), (b) clinical diagnosis, (c) MRI findings, (d) MRI technique, (e) MRI evaluation, (f) data analysis, and (g) results. In case of disagreements, consensus was reached through discussion.

#### Assessment of methodologic quality

The quality of the included articles was assessed with a set of quality criteria, based on the QUADAS tool [9]. The quality assessment was subdivided into four topics: (a) study sampling, (b) clinical diagnosis, (c) MRI evaluation, and (d) data analysis and results. Each item was rated from 0 to 2, resulting in a maximum of 8 points per article (Table 1). For longitudinal studies, the reporting on drop-out rates and reasons for dropping out were to be assessed.

**Table 1:** Quality scores for the articles on MRI and SpA fulfilling the inclusion criteria

	**Bollow *et al. *(1995) **[**10**]	**Klauser *et al. *(2004) **[**11**]	**Brandt *et al. *(2007) **[**12**]	**Weber *et al. *(2010) **[**13**]	**Wick *et al. *(2010) **[**14**]	**Kim *et al. *(2008) **[**15**]	**Weber *et al. *(2009) **[**16**]	**Bennett *et al. *(2009) **[**17**]	**Bennett *et al. *(2009) **[**18**]
**Study sampling (0-1)**	1	0	1	2	1	1	2	1	1

**Diagnosis (0-1)**	1	0	0	1	0	1	1	0	0

**MRI evaluation (0-1)**	0	0	0	2	2	1	2	2	1

**Data analysis and presentation of results (0-1)**	0	1	1	2	0	1	2	1	1

**Total (0-8)**	**2**	**1**	**2**	**7**	**3**	**4**	**7**	**4**	**3**

Diagnosis assessed on IBP or SpA diagnosed by criteria (best) or expert opinion (second best), same diagnosis performed on whole sample, diagnosis independent of MRI, diagnosis blinded for MRI, and reproducibility of diagnosis tested (inter- and intraexaminer reliability tested and reported). MRI evaluation assessed on clear definition of each relevant MRI finding, MRI blinded from diagnosis, reproducibility (inter- and intraexaminer reliability tested and reported), and short time between the MRI scan and diagnosis. Data analysis and presentation of results assessed on statistical significance test and confidence intervals reported where relevant and results presented in an understandable way. 0, Nonacceptable; 1, Reasonable; 2, Good; SpA, spondyloarthritis; IBP, inflammatory back pain.

The assessment was performed independently by the first and the third authors. Any disagreements in the assessments were settled by consensus and discussion with the second author. Articles with a quality score of more than 5 points were arbitrarily regarded as high-quality studies. Only results from high-quality studies were further reviewed.

### Statistical analysis and data synthesis

Sensitivity and specificity were retrieved from the article where possible and calculated from raw data when not reported in the article. The Wilson score method without continuity correction was used to calculate the 95% CI, if it were not reported in the article. Meta-analysis was planned to be performed on homogeneous high-quality studies, but was not performed because of insufficient data.

## Results

### Description of studies

**Figure 1:**
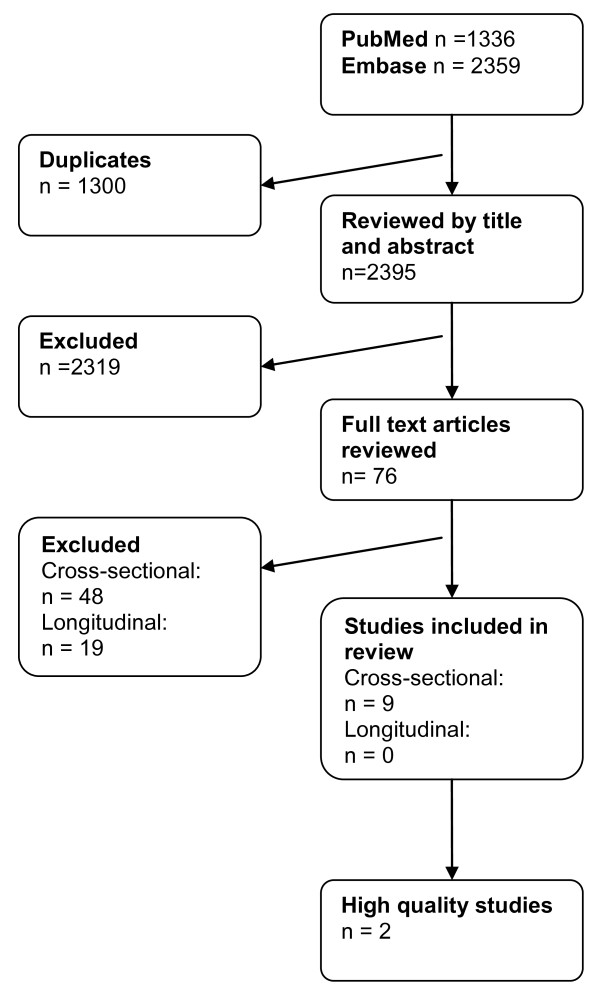
**Flow chart of the review process**.

In all, 1,336 articles were found in MEDLINE, and 2,359, in EMBASE. After elimination of duplicates, 2,395 articles were reviewed by title and abstract, and 76 articles were identified for assessment in full text. After full-text review, nine cross-sectional articles met the inclusion criteria [10-18] (Figure 1). No longitudinal studies met the inclusion criteria. Reasons for exclusion of articles reviewed in full text are presented in Table 2 for articles on cross-sectional studies and in Table 3 for articles on longitudinal studies.

**Table 2:** Reasons for excluding retrieved articles on cross-sectional studies

Study	Reasons for exclusion
Ahlstrom H, *et al. *(1990) [6]	< 20 controls
Algin O, *et al. *(2009) [25]	< 20 controls
Algin O, *et al. *(2010) [26]	< 20 controls
Baraliakos X, *et al. *(2005) [27]	No controls
Bejia I, *et al. *(2004) [28]	< 20 cases
Blum U, *et al. *(1996) [29]	Field strength, < 1.5 Tesla
Bochkova AG, *et al. *(2010) [30]	No controls
Bollow M, *et al. *(1993) [31]	Double publication on Bollow *et al. *[10]
Bollow M, *et al. *(1994) [32]	Double publication on Bollow *et al. *[10]
Bozgeyik Z, *et al. (*2008) [33]	No controls
Braun J, *et al. *(1994) [34]	< 20 controls
Braun J, *et al. *(1998) [35]	Reference standard (HLA-B27 status) not a clinical diagnosis
Bredella MA, *et al. *(2006) [36]	No controls
Chung H Y, *et al. *(2011) [37]	No controls
Docherty P, *et al. *(1992) [38]	< 20 controls
Friedburg H, *et al. *(1987) [39]	No controls
Gleeson TG, *et al. *(2005) [40]	< 20 cases
Gupta AD, *et al. *(2009) [41]	< 20 controls
Hanly JGJ, *et al. *(1994) [42]	< 20 controls
Heuft-Dorenbosch L, *et al. *(2006) [43]	Reference standard (plain radiographic) not a clinical diagnosis
Heuft-Dorenbosch L, *et al. *(2006) [44]	No controls
Heuft-Dorenbosch L, *et al. *(2007) [45]	No controls
Inanc N, *et al. *(2005) [46]	Reference standard (plain radiographic) not a clinical diagnosis
Jevtic V, *et al. *(1996) [47]	No controls
Liao Z, *et al. *(2009) [48]	No MRI of the control group
Liao Z, *et al. *(2011) [49]	No controls
Luukkainen RK, *et al. *(2007) [50]	No controls
Marc V, *et al. *(1997) [51]	< 20 controls
McNally EG, *et al. *(2001) [52]	< 20 cases
Muche B, *et al. *(2003) [53]	No controls
Murphey MD, *et al. *(1991) [54]	< 20 controls
Orchard TR, *et al. *(2009) [55]	< 20 cases
Peterova V *et al. *(2006) [56]	No controls
Puhakka KB, *et al. *(2003) [57]	No controls
Remy, *et al. *(1996) [58]	No controls
Rennie WJ, et al. (2009) [59]	< 20 controls
Rudwaleit M, *et al. *(2009) [60]	< 20 controls
Rudwaleit M, *et al. *(2009) [8]	Insufficient result presentation
Rudwaleit M, *et al. *(2009) [61]	Insufficient result presentation
Sreedhar C, *et al. *(2006) [62]	< 20 cases
Weber U, *et al. *(2007) [63]	< 20 controls
Weber U, *et al. *(2010) [64]	Double publication on Weber *et al. *[13]
Wecbach *et al. *(2009) [65]	No controls
Wienands K, *et al. *(1990) [66]	No controls
Williamson L, *et al. *(2004) [67]	Reference standard (orthopedic test) not a clinical diagnosis
Wittram C, *et al. *(1996) [68]	< 20 controls
Wittram C, *et al. *(1996) [69]	< 20 controls
Yu W, *et al. *(1998) [70]	< 20 controls

Studies may have met exclusion criteria other than those listed in the table. MRI, magnetic resonance imaging.

**Table 3:** Reasons for excluding retrieved articles on longitudinal studies

Study	Reasons for exclusion
Althoff CE, *et al. *(2009) [71]	Insufficient result presentation
Aydin SZ, *et al *(2011) [72]	< 20 controls
Baraliakos X, *et al. *(2008) [73]	No controls
Battafarano DF, *et al. *(1993) [74]	< 20 controls
Bennett AN, *et al. *(2008) [7]	No controls
Bigot J, *et al. *(1999) [75]	< 20 control
Brandt J, *et al. *(1999) [76]	No controls
Chiowchanwisawakit P, *et al. *(2011) [77]	No controls
Dougados M, *et al. *(2001) [78]	Insufficient result presentation
Hermann J, *et al. *(2009) [79]	Insufficient result presentation
Madsen K, *et al. *(2010) [80]	No controls
Macsymowych WP, *et al. *(2010) [81]	No controls
Marzo-Ortega H, *et al. *(2008) [82]	< 20 in each control group
Oostveen J, *et al. *(1999) [83]	No controls
Pedersen SJ, *et al. *(2011) [84]	No controls
Puhakka KB, *et al. *(2004) [85]	No controls
Remplik P, *et al. *(2005) [86]	< 20 cases
Shankar S, *et al. *(2009) [87]	Insufficient result presentation
Song IH, *et al. *(2011) [88]	No controls

Studies may have met exclusion criteria other than those listed in the table.

### Description of included articles

The nine articles meeting the inclusion criteria were based on eight study samples and were published between 1995 and 2010 [10-18]. Two studies were conducted on the same study population [17,18]. Seven of the nine articles were designed to address the diagnostic utility of MRI in the diagnosis of either AS, SpA, or IBP. One article compared ultrasound with MRI in the diagnosis of IBP [11], and one article evaluated referral recommendations [12]. Five studies reported results for the SIJ [10-14], and four, the results for the spine; one of the lumbar spine [15] and three of the whole spine [16-18]. The sample sizes varied from 85 to 187 participants. The diagnoses under evaluation were AS [10,12-16], SpA [10,12,14,17,18], and IBP [11,13,16]. The groups of noncases were no LBP [11,13,16-18] and/or noninflammatory low-back pain, such as NSLBP, degenerative arthritis, cancer, and LBP of unknown origin [10,12-15,17,18]. For further details of the included studies, see Table 4.

**Table 4:** Descriptive data for articles on MRI and SpA fulfilling the inclusion criteria

	Study type	Total number	Number of AS patients (DD)	Number of SpA patients (DD)	Number of IBP patients (DD)	Number of NILBP patients (DD)	Number of no LBP patients
**Sacroiliac joint**							

Bollow *et al. *(1995) [10]	Prospective case-control	125	36 (3.1 years)	36 (5.9 years)		53 (5.7 years)	
Klauser *et al. *(2004) [11]	Prospective case-control	133			103 (NR)		30
Brandt *et al. *(2007) [12]	Prospective cohort	158	32 (NR)	58 (NR)		68 (NR)	
Weber *et al. *(2010) [13]	Prospective case-control	187	75 (6.1 years)		27 (29 mo)	26 (NR)	59
Wick *et al. *(2010) [14]	Retrospective cohort	156	27 (NR)	101 (NR)		28 (NR)	

**Spine**							

Kim *et al. *(2008) [15]	Retrospective case control	104	52 (NR)			52 (NR)	
Weber *et al. *(2009) [16]	Prospective case-control	85	35 (8 years)		25 (10 mo)		35
Bennett *et al. *(2009) [17]	Retrospective cohort	185		64 (8.5 years)		110 (NR)	11
Bennett *et al. *(2009) [18]	Retrospective cohort	185		64 (8.5 years)		110 (NR)	11

MRI, magnetic resonance imaging; SpA, spondyloarthritis; AS, ankylosing spondylitis; DD, mean disease duration;

IBP, inflammatory back pain; NILBP, noninflammatory low-back pain; no LBP, no low-back pain; mo, months; NR, mean disease duration not reported.

All studies used an MRI scanner with a field strength of 1.5 Tesla. Four of the included articles had no description of the field strength, and the corresponding authors were therefore contacted for information of the field strength. Eight of the nine articles reported which MRI protocol was used [10,11,13-18]. For the SIJ, the most common sequences were T_1_-weighted spin-echo in combination with either a gadolinium sequence [10,11,14] or a Short Tau Inversion Recovery (STIR) sequence [13]. The slice orientation was either semicoronal or semiaxial or a combination of both. In the spine, the MRI protocol consisted of fewer sequences. Three articles reported the use of sagittal T_1_-weighted spin-echo and STIR [15,17,18], and one used only sagittal STIR [16]. None of the included studies evaluating the spine used contrast enhancement in the MRI protocol. For more details of the MRI protocols, see Table 5.

**Table 5:** Descriptive data for MRI technique used in the included articles

	Field strength	Sequence 1	Sequence 2	Sequence 3	Sequence 4	Sequence 5	Sequence 6
**Sacroiliac joint**							

Bollow *et al. *(1995) [10]	1.5 T	Semi axial T_1_w SE	Dynamic T_2_*w GRE, FLASH,	Dynamic T_2_*w GRE, FLASH, Gd			
Klauser *et al. *(2004) [11]	1.5 T	Semi axial T_1_w, SE	Semi axial T_2 _w TSE	Semi axial T_1_w, SE FS	Semi cor. T_1_w, SE	Semi cor. TIRM	Semi axial + Semi cor. T_1_w, SE, FS, Gd
Brandt *et al. *(2007) [12]	^a^1.5 T	NR					
Weber *et al. *(2010) [13]	^a^1.5 T	Semi cor. T_1 _w, SE	Semi cor. STIR				
Wick *et al. *(2010) [14]	1.5 T	Semi cor. T1 w, SE	Semi cor. TIRM	Semi cor. T_2_w, MEDIC FS	Semi cor. + semi axial T_1_w, SE, FS	Semi axial T_1_w, SE, FS, Gd	Semi axial T_1_w, SE, Gd

**Spine**							

Kim *et al. *(2008) [15]	1.5 T	Sagittal T_1_w SE	Sagittal T_2_w FSE				
Weber *et al. *(2009) [16]	1.5 T	Sagittal turbo STIR					
Bennett *et al. *(2009) [17]	^a^1.5 T	Sagittal T_1_, SE	Sagittal STIR				
Bennett *et al. *(2009) [18]	^a^1.5T	Sagittal T_1_w, SE	Sagittal STIR				

^a^Information on field strength not available in the articles. Data obtained by personal contact with authors. T, Tesla; w, weighted; SE, spin-echo; GRE, gradient-echo; FLASH, fast low-angle shot; Semi cor, semicoronal; Gd, gadolinium; TSE, turbo spin-echo; FS, fat saturated; TRIM, turbo inversion recovery magnitude; NR, not reported; STIR, short tau inversion recovery; MEDIC, multi echo data image combination; PD, proton density; FSE, fast spin echo.

### Assessment of methodologic quality

The quality score of the nine studies ranged from 1 to 7 points, with a mean score of 3.7. Two articles were rated more than 5 points and were considered of high quality [13,16], and three articles were rated less than 3 points [10-12] (Table 1).

In relation to the description of the study samples, five articles did not fully describe both cases and noncases with respect to age, gender, and diagnosis [11,14,15,17,18], three articles had no description of the sampling method used for the control groups [11,17,18], and for two of these [17,18], it was not clear whether these groups were included in the data analyses. Regarding the sampling methods used, one article reported the use of consecutive sampling [16]. The remaining eight either failed to report on this aspect or used convenience samples. Matching was performed adequately in relation to age and sex in four [10,13,15,16] of the five case-control studies. One study had a case group with a mean age that was more than 20 years older than that of the control group [11].

In relation to the diagnosis of the target condition, none of the nine articles tested the reproducibility of the clinical diagnosis. Diagnostic criteria were used as a reference standard in five articles [10,11,13,15,16], expert opinion in three articles [14,17,18], and both criteria and expert opinion in one article [12]. In four articles describing retrospective studies [14,15,17,18], the independence of MRI from the clinical diagnosis was unclear. In one prospective study [12], which aimed to test referral recommendations, MRI was recognized as part of the diagnosis for the group of preradiographic SpA, thereby confounding the association observed. In seven articles [10-12,14,15,17,18], inadequate or no reporting was made of blinding of the MRI results at the time of the clinical diagnosis.

In relation to the MRI evaluation, most studies had acceptable definitions of the MRI findings [13-18]. Five articles performed reproducibility tests of the MRI evaluation [13,14,16-18], and five studies blinded the evaluation for the diagnosis [13-17]. None of the nine articles reported the time interval between MRI and diagnosis.

In relation to the statistical analysis, one article failed to report fully the CI, *P *values, and/or raw data [10], and one article used an inappropriate statistical method for the types of data available [14]. Five articles had shortcomings in the way the results were presented [10,12,14,17,18], such as uncertainties in relation to which groups were included in the calculation of the specificity [17,18] and data primarily presented as graphics and per SIJ joint instead of per person [10].

### Association between MRI findings and the clinical diagnosis of SpA

Because of a substantial heterogeneity in the included studies regarding the methodologic quality, the MRI findings, the MRI technique used, and the regions under evaluation, it was not possible to perform a meta-analysis, and we decided not to report data systematically. Instead, a descriptive assessment of the results based on the extracted data was performed. One article of high quality was found that evaluated the SIJ [13], and another article of high quality was found that evaluated the spine [16], both of which were from the same research team. A summary of the extracted data from these two studies is presented in Tables 6 and 7.

**Table 6:** Associations between MRI finding in the SIJ and the diagnosis of AS and IBP

MRI findings	Sensitivity of MRI findings (95% CI)	Specificity of MRI findings (95% CI)	Cases, number		Controls, number	
			
			AS	IBP	NLSBP	No LBP
BMO	0.85 (0.76-0.92)	0.93 (0.84-0.97)	75			59
Erosions	0.91 (0.82-0.95)	0.98 (0.91-1.00)	75			59
Fat infiltration	0.91 (0.82-0.95)	0.86 (0.76-0.93)	75			59
Ankylosis	0.27 (0.18-0.38)	1.00 (0.94-1.00)	75			59
Global assessment	0.99	0.96	75			59

BMO	0.85 (0.76-0.92)	0.77 (0.58-0.89)	75		26	
Erosions	0.91 (0.82-0.95)	0.96 (0.81-0.99)	75		26	
Fat infiltration	0.91 (0.82-0.95)	0.85 (0.67-0.94)	75		26	
Ankylosis	0.26 (0.18-0.38)	1.00 (0.87-1.00)	75		26	
Global assessment	0.99	0.92	75		26	

BMO	0.67 (0.48-0.81)	0.93 (0.84-0.97)		27		59
Erosions	0.48 (0.31-0.66)	0.98 (0.91-1.00)		27		59
Fat infiltration	0.37 (0.22-0.56)	0.86 (0.76-0.93)		27		59
Global assessment	0.52	0.96		27		59

BMO	0.67 (0.48-0.81)	0.77 (0.58-0.89)		27	26	
Erosions	0.48 (0.31-0.66)	0.96 (0.81-0.99)		27	26	
Fat infiltration	0.37 (0.22-0.56)	0.85 (0.67-0.94)		27	26	
Global assessment	0.52	0.92		27	26	

Results from Weber *et al. *(2010). Sensitivity and specificity based on concordant observations of a minimum of two of five readers. 95% CI calculated from raw data. MRI, magnetic resonance imaging; SIJ, sacroiliac joint; AS, ankylosing spondylitis; IBP, inflammatory back pain; CI, confidence interval; NSLBP, nonspecific low-back pain; BMO, bone marrow edema; global assessments, 95% CI not possible to calculate, because of no raw data.

**Table 7:** Associations between MRI finding in the spine and the diagnosis of AS and IBP

MRI findings	Sensitivity of MRI findings (95% CI)	Specificity of MRI findings (95% CI)	Cases, number		Controls, number
			
			AS	IBP	No LBP
≥ 1 CIL	0.77 (0.61-0.88)	0.77 (0.61-0.88)	35		35
≥ 2 CIL	0.69 (0.52-0.81)	0.94 (0.81-0.98)	35		35
≥ 3 CIL	0.66 (0.49-0.79)	0.94 (0.81-0.98)	35		35
≥ 1 tCIL	0.69 (0.52-0.81)	0.83 (0.67-0.92)	35		35
≥ 2 tCIL	0.57 (0.41-0.72)	0.94 (0.81-0.98)	35		35
≥ 3 tCIL	0.46 (0.30-0.62)	0.97 (0.85-0.99)	35		35
≥ 1 lCIL	0.51 (0.36-0.67)	0.94 (0.81-0.98)	35		35
≥ 1 NIL	0.06 (0.02-0.19)	0.97 (0.85-0.99)	35		35
≥ 1 LIL	0.31 (0.19-0.48)	0.97 (0.85-0.99)	35		35
≥ 1 FIL/PIL	0.09 (0.03-0.22)	1.00 (0.90-1.00)	35		35

≥ 1 CIL	0.40 (0.23-0.59)	0.88 (0.70-0.96)		25	25
≥ 2 CIL	0.32 (0.17-0.52)	0.96 (0.80-0.99)		25	25
≥ 3 CIL	0.12 (0.04-0.30)	0.96 (0.80-0.99)		25	25
≥ 1 tCIL	0.32 (0.17-0.52)	0.88 (0.70-0.96)		25	25
≥ 2 tCIL	0.24 (0.11-0.43)	0.96 (0.80-0.99)		25	25
≥ 3 tCIL	0.12 (0.04-0.30)	0.96 (0.80-0.99)		25	25
≥ 1 lCIL	0.24 (0.11-0.43)	1.00 (0.87-1.00)		25	25
≥ 1 NIL	0.04 (0.01-0.20)	0.96 (0.80-0.99)		25	25
≥ 1 lCIL	0.12 (0.04-0.30)	0.96 (0.80-0.99)		25	25
≥ 1 FIL/PIL	0.00 (0.00-0.13)	1.00 (0.87-1.00)		25	25

Sensitivity and specificity based on concordant observations of three readers; results from Weber *et al. *(2009). AS, Ankylosing spondylitis; IBP, inflammatory back pain; no LBP, no low-back pain; CIL, vertebral corner inflammatory lesion; tCIL, thoracic CIL; lCIL lumbar CIL; NIL, vertebral noncorner inflammatory lesion; LIL, lateral inflammatory lesion; FIL/PIL, facet or other posterior element inflammatory lesion.

#### Sacroiliac joint

The study by Weber *et al. *from 2010 [13] included four groups in a prospective case-control study: 75 patients with AS included on the basis of the modified New York criteria [19], 27 patients with IBP based on the Calin Criteria [20] or the Berlin Criteria [21], 26 patients with NSLBP, and 59 healthy controls without back pain. The AS patients had a mean disease duration of 6.1 years, and IBP patients had a mean disease duration of 29 months. Disease duration for the NSLBP patients was not reported.

The MRI findings under evaluation were bone marrow edema (BMO), erosions, fat infiltration, global assessment of sacroiliitis, and ankylosis. Positive associations for BMO, erosions, fat infiltration, and global assessment of sacroiliitis were shown for both AS and IBP patients when compared with the NSLBP and no-LBP groups. Ankylosis was found in only the AS patients, in 20 of 75 patients (26.7%). In general, the highest combined values of sensitivity and specificity were found when comparing AS patients with the healthy controls, and the lowest values were found when comparing IBP with NSLBP (Table 6).

Interestingly, BMO lesions, as defined in the ASAS diagnostic criteria [8], were found to be relatively frequent in NSLBP and were reported in 6 (23.1%) of 26 subjects and less frequently in healthy controls, with 4 (6.8%) of 59 subjects.

#### Spine

The study by Weber *et al. *from 2009 [16] compared 35 patients with AS fulfilling the modified New York criteria [19], 25 patients with IBP, who fulfilled the Berlin criteria [21] and who also had one or more characteristic SpA features, and 35 healthy controls without back pain in a prospective case-control study. The AS patients had a mean disease duration of 8.0 years and the IBP patients had a mean disease duration of 10 months.

The MRI findings under evaluation were BMO lesions of varied number and location in the spine. When comparing AS patients with healthy controls, a number of positive associations were identified for BMO lesions in vertebral corners and in the lateral spine. When comparing IBP patients with healthy controls, the associations were seen to decrease (see Table 7 for details).

## Discussion

### Summary of main results

The results of the review show that a positive association exists between certain MRI findings in the axial skeleton and the diagnosis of both AS and IBP. However, these results are based on only two high-quality studies, and the amount of relevant data was insufficient to give a reliable quantification of this association.

### Implications for research

On the basis of the identified methodologic shortcomings of the included articles, this review highlights important issues that should be taken into consideration when conducting studies with the aim of evaluating the diagnostic utility of MRI in the diagnosis of SpA. The following issues will be discussed in the subsequent text: Size of the study sample, selecting the proper study population, sampling method of the study cohort, independence of MRI from the reference standard, reproducibility testing of both the reference standard and the MRI evaluation, and standardization of the MRI findings.

In this review, the inclusion criterion for the size of study samples was set to a minimum of 20 subjects per group. This was because a small sample size often gives imprecise estimates of diagnostic accuracy. For example, when the number of patients with SpA is 20, the two-sided 95% CI of a sensitivity of 80% is 58% to 92%. This is, however, still a very wide CI, and because wide CIs make it difficult to determine how informative the results of the study are, we would recommend that future studies use larger sample size. For example, if the number of patients with SpA is 200, the two-sided 95% CI of a sensitivity of 80% is 74% to 85%.

When choosing the study sample, it is crucial that an effort be made to make it as comparable to the population seen in a clinical setting as possible. Not surprisingly, the results from the two high-quality studies show that the association was stronger between patients with AS and asymptomatic people than the association between IBP patients and patients with NSLBP [13,16]. However, for the clinician, the challenge will often be differentiating between IBP and NSLBP. When investigating the diagnostic utility of MRI in SpA, it is therefore important that the study samples be representative of "real" patient populations, instead of comparing patients with long-lasting disease and asymptomatic persons. Furthermore, one of the high-quality studies reports inflammatory MRI findings in the SIJ to be relatively common in patients with NSLBP [13]. Because the specificity is estimated entirely from results from the group of noncases or controls, this, additionally, underlines the need for further evaluation of the presence of MRI findings also in patients with NSLBP.

The sampling methods used in the included studies were either insufficient or not fully described in the majority of the studies, which makes it difficult to preclude selection bias. It is mandatory when conducting studies on diagnostic accuracy that both the sampling method and the study sample are well described, that the selection of study participants is performed systematically and unselectively, and, for case-control studies, that the two groups are comparable except for the target condition. Furthermore, in some of the articles identified in this review, the importance of the controls seemed to be underestimated, even though the sampling and description of the controls are just as important as the sampling and description of the cases.

That no objective gold standard exists for the diagnosis of SpA, which is instead based on a composite clinical presentation, constrains the possibilities for the evaluation of the diagnostic accuracy of MRI. However, it is indisputable that when evaluating a diagnostic test, independent evaluation of the index test and the reference standard is essential. When MRI is used in the decision making of the clinical diagnosis, as seen in more than half of the included studies, incorporation bias is highly likely. This was predominantly a problem in the included retrospective studies. The fact that MRI is widely used in the diagnosis of SpA restricts the possibilities of conducting meaningful retrospective studies on the diagnostic accuracy of MRI.

One way to strengthen the reliability of the diagnosis is to test the reproducibility of the selected reference standard. However, none of the nine included articles tested the reproducibility of the clinical diagnosis. Another way to strengthen the reliability of the diagnosis would be to include a longitudinal aspect and to follow the development of more-advanced disease stages to confirm the diagnosis. Even though several longitudinal studies were identified in this review, none met the inclusion criteria, usually because either no or too few controls were used.

Several activity scores for inflammatory and structural MRI findings have been developed; however, no international consensus exists about the definition of diagnostic MRI findings, which was a factor in our review. In general, the included studies contained definitions of the MRI findings. However, little homogeneity existed in these definitions, and only five of the nine articles reported testing of the reproducibility of the MRI evaluation. Before testing the diagnostic utility of an MRI finding, it is necessary to define the MRI finding and further to present acceptably reproducible test results. Furthermore, an experienced musculoskeletal radiologist should be part of the conduct and testing of the evaluation protocol. An international agreement on definitions of different MRI findings and a threshold minimal reproducibility would facilitate both the translation from research to clinical practice and a comparison with future research results.

In summary, the optimal design for future studies that investigate the utility of MRI in the early diagnosis of SpA is a large longitudinal clinical cohort study that recruits participants consecutively and includes patients with NSLBP. Furthermore, a well-defined clinical diagnosis based on consensus criteria and blinded for MRI should be used as a reference standard. Similarly, a validated MRI evaluation with clear definitions blinded to the clinical information should be used for the MRI evaluation.

### Application of findings to clinical practice

The literature search associated with the current review revealed no other systematic critical review on the utility of MRI in the diagnosis of SpA. However, a large number of commentaries and narrative reviews with or without transparent search strategies have been published, in which it seems that the majority report a positive view of the value of MRI in the diagnosis of SpA. The obvious need for optimization of the diagnostic process of SpA may have contributed to the positive perception that has developed regarding MRI as a tool in the SpA diagnosis. The fact that MRI of the SIJ is included in the new diagnostic criteria developed by ASAS [8] has led to further optimism in relation to the diagnostic process of SpA. However, on the basis of the results from the current review, the limited number of high-quality studies on the diagnostic utility of MRI in SpA should preclude MRI being seen as the new gold standard in the diagnosis of SpA. If inflammatory and structural MRI findings are interpreted as being more specific for SpA than the research can support, a real risk exists of SpA being overdiagnosed.

As mentioned earlier, to be able to make a reasonable translation of research results to clinical practice, the representativeness of the study sample to clinical practice and the standardization and validation of the MRI evaluation is important. However, it also is essential that the MRI protocols used in the studies are relatively easy to implement and constitute the least health risk for the patients. Three of the studies included in this review used gadolinium contrast-enhanced sequences. However, for future studies, the necessity of gadolinium should be carefully considered, because other techniques, such as STIR, have shown a similar capability of visualizing BMO [22,23], and the use of contrast is more invasive, expensive, and time consuming than is a noncontrast method. Furthermore, a small but serious risk of contrast medium-induced nephrotoxicity exists [24], which also must be taken into consideration.

### Strengths and weaknesses of the review

The strength of the current review is the systematic search method and the rigorous assessment of the methodologic quality of the included studies. To our knowledge, this review is the first to give an overview of the level of evidence of the diagnostic utility of MRI, including a quality assessment.

The weaknesses of the review are first, the possibility of relevant articles not being included because of the language limitations, and second, the limited number of databases used in the search. Furthermore, it might be argued that the threshold for a minimal study sample size was either too low or too high. However, it seems reasonable to reduce the width of the 95% CI by not setting the cut-point too low; in contrast, increasing the cut-point for sample size further would have greatly decreased the number of relevant articles that could be included.

## Conclusion

Bone marrow edema, erosions, fat infiltrations, global assessment of sacroiliitis, and ankylosis on MRI were found to be associated with SpA. However, because only two high-quality studies were identified, it was not possible to perform a meta-analytic quantification of the association. The current systematic critical review illustrates that only limited evidence exists of the utility of MRI in the diagnosis of SpA. However, MRI is already widely used in clinical practice, and therefore, a strong need exists for more high-quality studies, cross-sectional as well as longitudinal, without the shortcomings of the previous work identified in this review. In the meantime, caution is needed to avoid overdiagnosing SpA.

## Abbreviations

AS: ankylosing spondylitis; ASAS: Assessment of SpondyloArthritis international Society; BMO: bone marrow edema; CI: confidence interval; IBP: inflammatory back pain; MRI: magnetic resonance imaging; NSLBP: nonspecific low-back pain; SIJ: sacroiliac joint; SpA: spondyloarthritis.

## Competing interests

The authors declare that they have no competing interests.

## Authors' contributions

BA conducted the collection, analyses, and interpretation of the data, and drafted the manuscript. TSJ participated in the collection, interpretation and analyses of the data and helped to draft the manuscript. CLY participated in the interpretation of data and provided editorial assistance. All authors were an integral part of the study design and critically reviewed, contributed to, and approved the final manuscript.
